# Developing a Semi-Supervised Approach Using a PU-Learning-Based Data Augmentation Strategy for Multitarget Drug Discovery

**DOI:** 10.3390/ijms25158239

**Published:** 2024-07-28

**Authors:** Yang Hao, Bo Li, Daiyun Huang, Sijin Wu, Tianjun Wang, Lei Fu, Xin Liu

**Affiliations:** 1 Wisdom Lake Academy of Pharmacy, Xi’an Jiaotong-Liverpool University, Suzhou 215123, China; yang.hao20@student.xjtlu.edu.cn (Y.H.); bo.li21@student.xjtlu.edu.cn (B.L.); sijin.wu@xjtlu.edu.cn (S.W.); tianjun.wang19@student.xjtlu.edu.cn (T.W.); lei.fu@xjtlu.edu.cn (L.F.); 2 Institute of Systems, Molecular and Integrative Biology, University of Liverpool, Liverpool L69 7ZX, UK; 3 School of Life Sciences, Fudan University, Shanghai 200092, China

**Keywords:** Support Vector Machine (SVM), multitarget drug, PU-learning, virtual screening

## Abstract

Multifactorial diseases demand therapeutics that can modulate multiple targets for enhanced safety and efficacy, yet the clinical approval of multitarget drugs remains rare. The integration of machine learning (ML) and deep learning (DL) in drug discovery has revolutionized virtual screening. This study investigates the synergy between ML/DL methodologies, molecular representations, and data augmentation strategies. Notably, we found that SVM can match or even surpass the performance of state-of-the-art DL methods. However, conventional data augmentation often involves a trade-off between the true positive rate and false positive rate. To address this, we introduce Negative-Augmented PU-bagging (NAPU-bagging) SVM, a novel semi-supervised learning framework. By leveraging ensemble SVM classifiers trained on resampled bags containing positive, negative, and unlabeled data, our approach is capable of managing false positive rates while maintaining high recall rates. We applied this method to the identification of multitarget-directed ligands (MTDLs), where high recall rates are critical for compiling a list of interaction candidate compounds. Case studies demonstrate that NAPU-bagging SVM can identify structurally novel MTDL hits for ALK-EGFR with favorable docking scores and binding modes, as well as pan-agonists for dopamine receptors. The NAPU-bagging SVM methodology should serve as a promising avenue to virtual screening, especially for the discovery of MTDLs.

## 1. Introduction

Drug discovery today mainly focuses on designing ligands with maximum selectivity to act on individual drug targets, with the aim of reducing the risk of off-target related side effects. However, many complex diseases, such as cancer [1,2], neurodegenerative diseases [3,4], cardiovascular diseases [5], and infection [6], involve multiple progression-relevant targets and/or signaling pathways. For these multifactorial diseases, relying on a single medicine to modulate the biological function of a solitary target might be inadequate to achieve satisfactory efficacy, despite the best research efforts. Hence, there is a growing interest in developing multitarget agents capable of simultaneously intervening in multiple receptors, with the goal of enhancing therapeutic efficacy and ensuring safety [7,8,9].

There are two approaches to multitarget therapeutics: drug combination, and multitarget-directed ligands (MTDLs) [10]. While the former approach may offer greater dose flexibility and lower clinical trial costs, it often grapples with treatment complexity, drug side effects, pharmacokinetic intricacies, and drug–drug interactions [11,12]. Conversely, well-designed and finely balanced MTDLs have the potential to circumvent these challenges, all the while capitalizing on the benefits of multitarget therapeutics, such as synergistic effects [13,14,15]. In essence, strategic combinations of targets can enable the use of lower drug doses to achieve sufficient therapeutic effects, as partial modulation of synergistically acting targets may be satisfactory for achieving full therapeutic efficacy. Consequently, this approach can mitigate drug side effects and widen therapeutic windows [16].

To date, several remarkable MTDLs have been identified, developed, and further approved for clinical use. A significant portion of these discoveries, such as the multitarget kinase inhibitor imatinib, occurred serendipitously, while others primarily stem from knowledge-based approaches in which scaffolds from different active molecules with known activity against a particular target are combined [17,18]. An alternative and potentially more practical strategy is the screening of natural compounds that can directly modulate multiple targets, offering an effective avenue for discovering MTDLs with improved bioavailability [19,20].

In recent years, numerous promising virtual screening methods based on machine learning (ML) or deep learning (DL) have been proposed [21,22,23,24], shaping the landscape of artificial intelligence in both single-target drug and MTDL discovery. Despite the promising performance that these methods have claimed, the design and choice of ML/DL architectures often appears arbitrary [25,26]. The existing literature provides limited comparisons on the superiority of traditional ML and DL algorithms, yielding inconclusive findings [27,28,29,30]. A recent impartial viewpoint suggests that no singular learning approach surpassed the others [27,28]. This also underscores, alongside the utilized modeling techniques, the significance of meticulous curation of training data [26,29] and the selection of molecular representations [30].

For any chosen ML or DL algorithm, the immediate and crucial next step is the selection of an appropriate molecular representation method, which largely contributes to the quality and efficiency of these compound activity prediction models. Many molecular descriptors and fingerprints have been derived from human expert knowledge to provide a comprehensive representation of the constitutional, physicochemical, topological, structural, and substructural features of molecules [31,32]. Furthermore, the utilization of neural network architectures for learning representations from graph-structured data stands as a cutting-edge strategy and has led to substantial enhancements in the learning of pharmaceutical and physicochemical properties [33]. Unfortunately, the evaluation of how to select the most suitable molecular representation method for the chosen ML/DL algorithm, or which combination of representation methods and ML/DL algorithms can achieve superior predictive performance, has rarely been systematically assessed in prior studies, leading to a lack of definitive answers.

In addition, the accuracy and robustness of these ML/DL-based compound activity prediction methods heavily rely on the training dataset, where both negative and positive samples play equally vital roles. Bioassay data obtained from the high-throughput screening are usually imbalanced, with a significantly larger number of active compounds compared to inactive compounds [34] (Supplementary Tables S1 and S2). Consequently, the scarcity of negative data can lead to unexpected high false positive rates [35]. To mitigate the issue induced by data sparsity, considerable efforts have been directed towards data augmentation, which mainly entails the random generation of negative samples from the entire chemical space [36,37] or from a selection of clustered regions [38]. However, the chemical space used for generating negative samples lacks experimental assay values (unlabeled data), potentially leading to the inclusion of active (positive) compounds in the generated negative samples, thereby compromising the credibility of the ML/DL prediction models.

In this context, positive unlabeled (PU) learning, a valuable semi-supervised learning algorithm that leverages unlabeled data with diverse features and potential actives, improves classification effectiveness in situations where negative samples are absent. It achieves this by enhancing the reliable extraction of negative samples from unlabeled pools, and it has shown promising performance in real-world applications within the domain of drug discovery [39,40,41]. PU learning offers the benefit of expanding the sample size and information reservoir, alleviating the impact of data sparsity and distribution shift, thus boosting the model’s capacity for generalization.

Based on PU learning, in this study, we aim to address three key questions through a series of experiments focusing on virtual screening of multitarget kinase inhibitors:

1. Do DL methods consistently outperform traditional ML methods, specifically identifying the best-performing ML/DL method for the compound activity prediction scenario?
2. Which molecular representation method(s) are most appropriate for the selected ML/DL method?
3. How can unlabeled or putative negative data be effectively utilized to enhance the true positive rate while keeping the false positive rate of virtual screening models within acceptable limits?

To address these questions, we meticulously compared various ML/DL methods, including Support Vector Machine (SVM) and ten prevalent neural network architectures, along with a range of molecular representation techniques encompassing fingerprints, physicochemical descriptors, and neural networks learning from molecular graphs. Following the evaluation, SVM and ECFP4 emerged as superior performers among the ML/DL and representation methods, prompting their selection as the baseline for further assessment of data augmentation strategies.

Subsequently, we delved into exploring different data augmentation strategies, leading to the development of a novel semi-supervised learning framework named Negative-Augmented PU-bagging (NAPU-bagging) SVM. Our comparative analyses revealed that conventional data augmentation techniques often entail a trade-off between the true positive rate and the false positive rate. In contrast, NAPU-bagging SVM is capable of enhancing the true positive rate without necessitating a sacrifice in the false positive rate. Recognizing its potential in virtual screening, particularly in the context of MTDL discovery, where a high true positive rate is crucial for identifying potential interactions between candidates of multiple targets, we effectively applied this framework. It was instrumental in identifying potential MTDLs targeting the epidermal growth factor receptor (EGFR) and ALK tyrosine kinase receptor (ALK) in non-small-cell lung cancer (NSLC), as well as pan-agonists for dopamine receptors (DRD1–DRD5) for the treatment of neurodegenerative diseases. The outcomes underscored the NAPU-bagging SVM’s proficiency in managing false positive rates in virtual screening while maintaining high recall rates, as well as its ability to unveil structurally novel hit molecules. In summary, the NAPU-bagging SVM presents a robust semi-supervised learning framework for active compound screening, thereby advancing the realm of poly-pharmacology therapy for complex diseases.

## 2. Results and Discussion

### 2.1. SVM Outperforms DL-Based Drug–Target Potency Prediction Models

To address the first question, i.e., to identify the best-performing ML/DL method for compound–protein interaction prediction, we initially selected eleven ML/DL methods for a comprehensive comparison, including SVM and ten other popular DL-based sequence-to-drug models. SVM was specifically chosen for its effectiveness in handling imbalanced data with its tunable hyperparameter misclassification penalty parameter C and maximum-margin hyperplane. Moreover, SVM has outperformed other traditional ML methods on diverse datasets in other similar evaluations [42]. Additionally, we chose SVM for its consistently superior performance in regression tasks [43], which opens the possibility of extending the interaction probability prediction model in this study to interaction activity prediction. Presently, over forty significant DL-based models for predicting drug–target potency have been developed and have demonstrated promising performance. Among these models, we selected ten, including DeepDTA [44], DeepConv-DTI [37], TransformerCPI [45], VQA-seq [46], MolTrans [47], GraphDTA [48], MGraphDTA [49], HyperAttentionDTI [50], DrugBAN [51], and TransformerCPI2.0 [52]. These models provide varied protein featurization, compound representation, and network frameworks for potency learning, enabling a comprehensive comparison.

**Figure 1:**
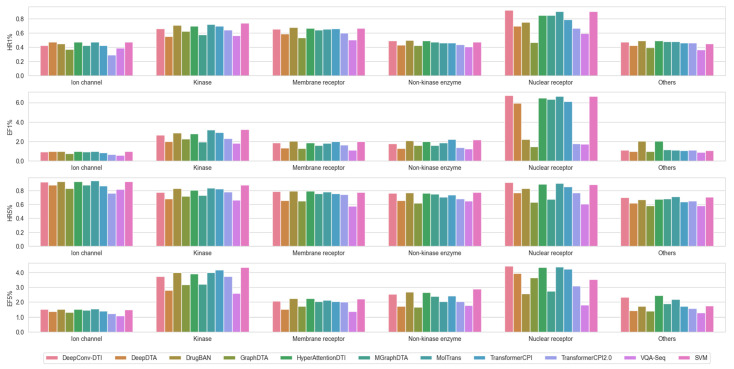
Comparison of model performance of the 11 ML/DL methods based on hit rates and enrichment factors across ChEMBL targets (categorized by target family). Hit rate (HR): HR1% and HR5% represent the percentage of actual active compounds among the top 1% and 5% of predicted hits, respectively. Enrichment factor (EF): EF1% and EF5% are calculated as the ratio of HR1% and HR5% to the overall ratio of active compounds to the total number of compounds in the library; for both metrics, the higher the better.

In particular, SVM trained on Morgan fingerprints and the ten chosen DL-based models were applied to ChEMBL targets with a minimum of 680 compound entries containing bioactivity data (see Supplementary Table S3 for more details). This threshold was chosen to maintain 80% of the total sample for comparison, and to ensure statistical significance for each target test set size. The results showed that SVM demonstrated superior or at least comparable performance to the ten DL-based models (Figure 1). Therefore, SVM served as the baseline for the subsequent evaluation of molecular representation and data augmentation strategies.

### 2.2. ECFP4 Outperformed Other Compound Representation Methods

The efficacy of ML/DL-based compound–protein interaction prediction methods greatly hinges on the selection of input features. Therefore, for the chosen ML/DL method, the next crucial step is selecting an appropriate representation for the learned objects, particularly small molecules in this context. In this study, we considered 7 topological path-based fingerprints (including ECFP4, AtomPairFP, TorsionFP, RDkitFP, AvalonFP, MHFP, and MAP4), 2 pharmacophore-based fingerprints (including PharmacoErGFP and PharmacoPFP), 3 substructure-key SMARTS-based fingerprints (including PubChemFP, MACCSFP, and EstateFP), and a compilation of 13 constitutional, physicochemical, and topological descriptors (referred to as “descriptors” for simplicity). Furthermore, recent advancements in developing neural network architectures capable of learning representations from molecular graphs have demonstrated superiority over traditional methods relying on molecular descriptors and fingerprint features [33]. As a result, one model from this category, AttentiveFP, was also included in the assessment, using the same training and evaluation datasets.

**Figure 2:**
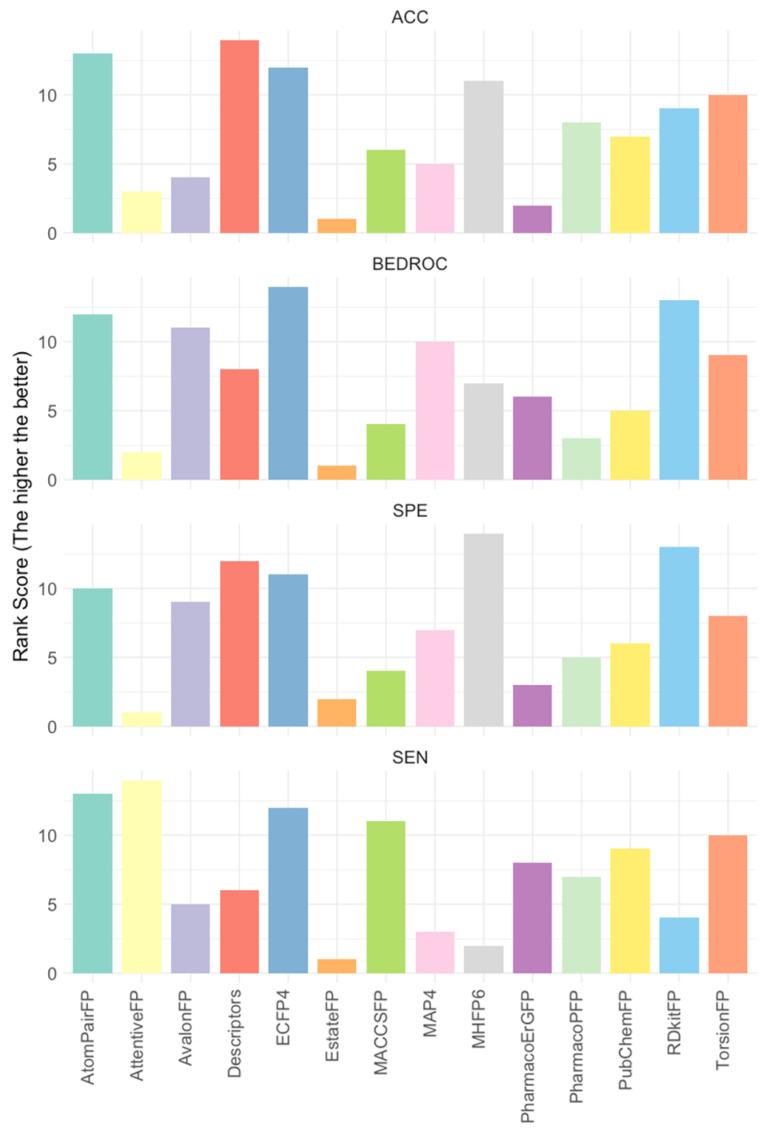
The average rank of 14 features across 17 evaluation datasets. Rankings were based on performance metrics including accuracy (ACC), BEDROC, specificity (SPE, 1—false positive rate), and sensitivity (SEN, true positive rate). The higher values indicate more favorable rankings and superior performance; 17 evaluation datasets: 6 kinase targets × 3 evaluation datasets (ChEMBL v32, ExCAPE, and DrugBank), where VEGFR1 has no ExCAPE dataset.

Given the significant variance in positive-to-negative ratios across datasets (see Supplementary Table S4 for more details), direct comparisons of evaluation metrics such as false positive and true positive rates across datasets are not feasible. To address this, we calculated feature ranks for each evaluation dataset, which were then averaged to determine the final rank for each feature. Key evaluation metrics including accuracy, BEDROC, false positive rate, and true positive rate were considered. The ridge lines in Figure 2 illustrate the averaged ranks for each feature under each metric, with higher ranks indicating superior performance (see Supplementary Table S5). Notably, ECFP4 consistently ranked at or near the top across all four evaluation metrics. As a result, ECFP4 was utilized as the molecular representation method for all subsequent data augmentation strategies.

### 2.3. Data Augmentation Strategies for Constructing Semi-Supervised Learning Models

**Figure 3:**
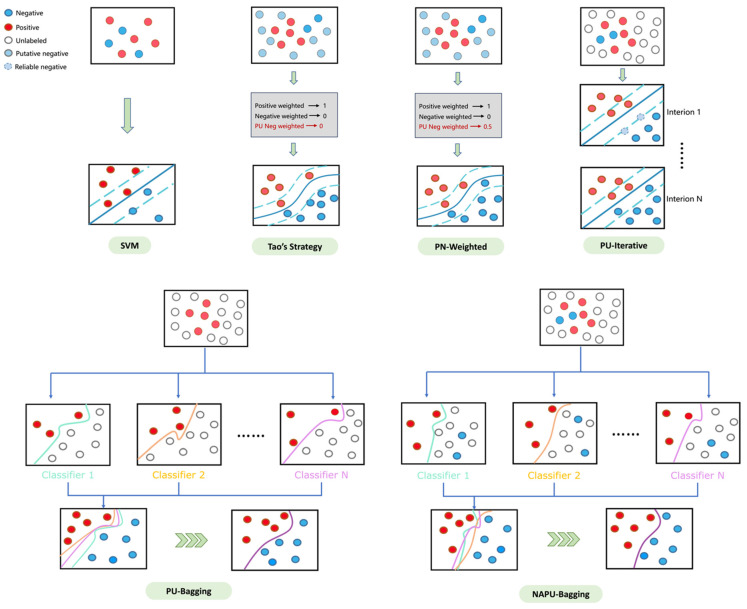
An illustrative diagram of various data augmentation strategies.

Developing ML/DL-based compound–protein interaction prediction models for virtual screening often faces challenges due to limited data availability on active and inactive molecules relevant to the target, as well as a lack of compound diversity covering the entire chemical space [53] (see Supplementary Tables S6 and S7). These data constraints can significantly hamper classification accuracy and generalization capabilities, particularly impacting the optimal classification boundaries, most notably for SVM algorithms. To address these limitations, we implemented diverse data augmentation strategies (Figure 3). To assess the effectiveness of these strategies, we conducted evaluations on six tumor-related targets, including ALK, MET, EGFR, MAPK1, FGFR1, and VEGFR1.

True positive and negative compounds relevant to these targets were utilized to establish the SVM model as the *baseline*. Furthermore, we integrated the generated putative negative sets for each target during training to implement *Tao’s strategy* [38]. The results were consistent with Tao’s findings [38], demonstrating the effectiveness of including putative negatives in reducing FPRs in virtual screening (Table 1). However, this strategy also introduces a trade-off by sacrificing a portion of the true positive rate. While this trade-off may be acceptable for single-target drug screening, it becomes more critical in multitarget drug screening scenarios, where the loss in the true positive rate from single-target screening can have a cascading effect and significantly impact the outcomes of multitarget screening, resulting in an extremely low yield. Therefore, a refined strategy is required to achieve an optimal balance, ensuring enhanced recall while effectively managing the FPR.

*Tao’s strategy*, which generates putative negative sets from molecular clusters without known active compounds, enhances the confidence in these putative negatives. However, clusters containing known active compounds may also include inactive compounds, leading to the presence of structurally similar compounds with vastly different activities, known as the “activity cliff” phenomenon. Therefore, building upon *Tao’s strategy*, we opted to utilize unlabeled data derived from clustered regions spanning the entire chemical space instead of using putative negative sets to improve the model’s recall rate. Additionally, clusters without known active compounds may still contain active compounds, suggesting that the credibility of negative sets generated from these clusters should be lower compared to true negative sets. To address this, we explored two strategies: first, assigning weights below 1 to the incorporated unlabeled data (0.5 in this study, referred to as the *PN-weighted strategy*); second, utilizing true positive and negative sets as the initial training set, where compounds predicted as negative in the unlabeled sets are iteratively included in subsequent training rounds until no new compounds are predicted as negative in the unbalanced sets, aiming to refine the boundary of the classifier (referred to as the *PU-iterative strategy*). The results (Table 1) suggest that these two strategies are notably effective in managing the false positive rate. However, there was an unexpected decrease in the recall rate compared to *Tao’s strategy*, possibly attributed to the unavoidable overfitting issue resulting from the substantial increase in negative samples in the training set.

Bagging-based classification has shown various advantages, such as preventing overfitting and reducing estimation variance, making it particularly valuable in the context of PU learning [54]. Therefore, to alleviate the issue of decreased recall caused by overfitting in both the *PN-weighted* and *PU-iterative* strategies, we explored an inductive bagging-based PU learning (*PU-bagging*) strategy. This strategy involves utilizing bootstrap aggregation to create multiple resampled bags from the training data, with each bag consisting of all true positive samples and randomly sampled unlabeled instances at a fixed ratio (1:1 in this study). An ensemble of SVM classifiers is trained on each bag of samples to distinguish positive samples from unlabeled ones. The final prediction is aggregated from the collective outputs of the ensemble of classifiers. As shown in Table 1, the *PU-bagging* SVM achieved significantly higher sensitivity/recall on the validation sets compared to previous strategies, consistent with observations in the existing literature [55,56]. This outcome further underscores the efficacy of the bagging technique in augmenting the recall/sensitivity of classifiers.

### 2.4. Developing a Novel Negative-Augmented PU-Bagging (NAPU-Bagging) SVM for Enhanced Virtual Screening

While enhancing the recall/sensitivity is a desirable trait for virtual screening, the *PU-bagging* SVM encountered a reduction in specificity, potentially impacting subsequent virtual screening processes by increasing the occurrence of false positives. Our hypothesis is that despite the bagging technique demonstrating heightened recall/sensitivity through mitigated overfitting by training ensemble classifiers on diverse samples [57], the presence of positive contamination in the unlabeled data may still introduce noise for each bootstrap classifier in establishing a decision boundary. Leveraging the available yet limited true negative samples for each target could potentially alleviate this issue. In order to maintain the elevated recall/sensitivity of the strategy while moderating its FPR to a satisfactory level, we propose a novel Negative-Augmented PU-bagging (*NAPU-bagging*) SVM approach. This approach enriches each bag by resampling true negative samples alongside the original true positive and unlabeled samples, following a predefined ratio (e.g., 1:0.2:0.8 for positive [P], negative [N], and unlabeled [U], respectively), and merging the negative samples with the unlabeled samples into a single class. Our refined data augmentation strategy ensures a controlled and substantial presence of “strong” negatives in each bag, aligning the feature distribution of unlabeled bags more closely with that of non-positive instances, promoting clearer class differentiation and reduced misclassification.

**Figure 4:**
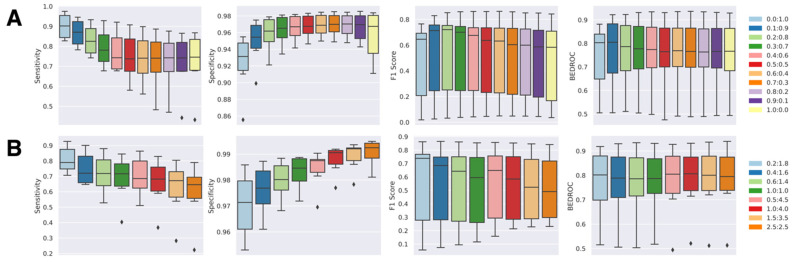
Performance of NAPU-bagging SVM on kinase target test sets: (**A**) Varying negative-to-unlabeled ratios, maintaining a bag size equal to the number of positive samples (1.0 times). (**B**) Selected negative-to-unlabeled ratios, maintaining a bag size equal to 2.0 times or 5.0 times the number of positive samples.

The classification boundary of the *NAPU-bagging* SVM was then optimized by adjusting the bag size and the sampling proportions of negative samples alongside the original positive and unlabeled samples, aiming for the best predictive performance. We trained *NAPU-bagging* classifiers with different P:N:U ratios by fixing P at 1.0 and tested their recall/sensitivity (SEN), specificity (SPE), F1-score, and BEDROC_80.5_ metrics on validation sets for the six kinase targets (Figure 4). For optimal parameter selection, we adhered to the principle of achieving high sensitivity while maintaining sufficiently high specificity. Initially, we maintained the bag size consistent with the number of P samples and varied the N:U ratio from 0.0:1.0 (equivalent to the *PU-bagging* SVM, which only uses true positive and unlabeled samples) to 1.0:0.0, which revealed a clear trade-off between sensitivity and specificity—higher specificity accompanied by lower sensitivity was observed with more true negative samples used for augmentation in bagging (Figure 4A).

Subsequently, we adjusted the bag size by setting the bag size at two or five times the number of true positive samples, ensuring that it remained within the limits defined by the true negative sample size for each target. This modification allowed us to experiment with different N:U ratios. Interestingly, we found that this adjustment resulted in further increased specificity and reduced sensitivity compared to scenarios where the bag size was equal to the number of true positive samples (Figure 4B). Based on these observations, we concluded that setting the bag size equal to the number of true positive samples, along with establishing a P:N:U ratio of 1:0.4:4.5, achieved a well-balanced performance across all evaluation metrics (Table 1). Consequently, we determined that these parameters would serve as the final configuration for the predictive models of the six selected targets.

To further validate the virtual screening performance of the optimized models, we evaluated them using the 0.46M Enamine HLL-460 library as the virtual screening set. The results indicated that the models identified a very small percentage of compounds as virtual hits for each target: 12 (0.0026%), 25 (0.0054%), 29 (0.0063%), 336 (0.073%), 380 (0.0826%), and 668 (0.1452%) for MET, FGFR1, ALK, EGFR, VEGFR1, and MAPK1, respectively. It is important to note that even if all of these virtual hits were false positives, the maximum false hit rate would only range from 0.0025% to 0.1452%. Hence, we can confidently state that the proposed NAPU-bagging SVM approach is highly capable of efficiently searching extensive chemical libraries, with an exceptionally low false hit rate.

Nonetheless, we also observed diminished performance of NAPU-bagging SVM on certain tested targets, particularly MAPK1 and VEGFR1, especially in terms of F1-score (Table 1). This can be attributed to distinct factors. As depicted in Supplementary Tables S4 and S6, MAPK1 stands out as one of the kinases with extensive bioactivity data in available databases, especially regarding its negative compounds (14,454 compounds from 8687 distinct clusters). Given that NAPU-bagging SVM is primarily designed to address the prevalent issue of insufficient volume and/or diversity in negative data, which is not a problem for MAPK1, its lack of improvement or even diminished performance on MAPK1 is logically consistent. In the case of VEGFR1, although NAPU-bagging SVM slightly outperforms the baseline SVM model across all metrics, the unsatisfactory F1-score could be attributed to the very limited number of active samples in the test datasets, with a ratio of 31 positive to 8906 negative samples. This skewed distribution means that even a single misclassification of an active sample can significantly skew the evaluation metrics.

### 2.5. NAPU-Bagging SVM in Virtual Screening of Novel Dual-TKIs for NCSLC

Somatic activating mutations in EGFR and chromosomal rearrangements involving ALK are the leading oncogenic drivers in non-small-cell lung cancer (NSCLC) [58]. The treatment landscape for NSCLC has advanced significantly with the introduction of multiple EGFR and ALK tyrosine kinase inhibitors (TKIs) as highly effective agents. However, the emergence of acquired resistance poses a substantial challenge [48]. While combination therapies are an alternative approach to combat acquired resistance, they often encounter complexities related to differing bioavailability, pharmacokinetics, metabolism, and drug interactions [59]. Utilizing dual- or multitarget drugs, which integrate multiple biological actions in a single compound, enables the simultaneous targeting of multiple pathways. This simplifies treatment regimens, minimizes potential drug interactions [59], and therefore offers a more effective solution for acquired resistance with improved safety measures.

As of now, only one dual-TKI targeting EGFR and ALK, Brigatinib [60], has received clinical approval (see Supplementary Table S8 for its affinity data). This section aims to utilize the refined NAPU-bagging SVM models to identify new potential dual-TKIs for EGFR-ALK, offering an alternative therapeutic approach for NSCLC. Additionally, this case study serves to assess the effectiveness of the *NAPU-bagging* strategy in virtual screening for the discovery of MTDLs.

**Figure 5:**
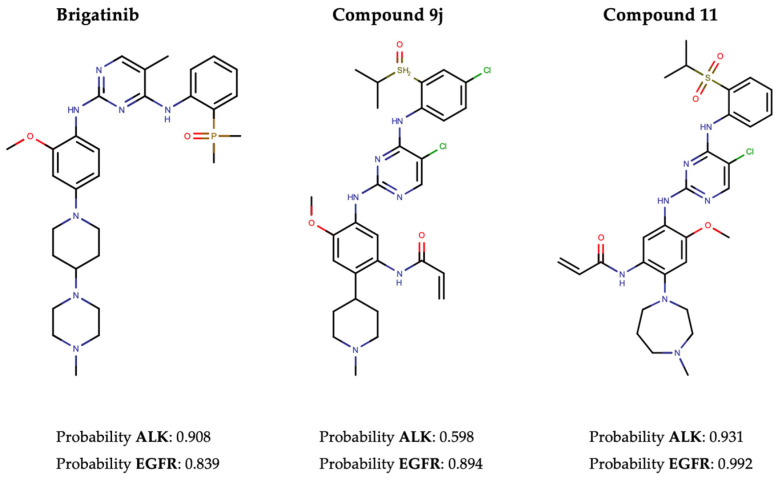
The structures and predicted probability values of three known dual ALK-EGFR inhibitors.

In pursuit of this, we first conducted a screening of the Therapeutic Target Database (TTD) to identify 100 approved TKIs, TKIs in clinical trials, and preclinical TKIs targeting EGFR, along with 18 for ALK (Supplementary Tables S9 and S10). The performance evaluation primarily focused on three metrics: recall, hit rate, and enrichment factor (EF). The results revealed recall rates of 86.4% and 55.4%, and EF1% of 32.98 and 77.13, for ALK and EGFR, respectively. In addition, Brigatinib, along with two other recently developed dual ALK-EGFR inhibitors (Compound 9j [61] and Compound 11 [62]), was successfully identified by our NAPU-bagging SVM among the hit ALK-EGFR dual-TKIs (Figure 5).

**Figure 6:**
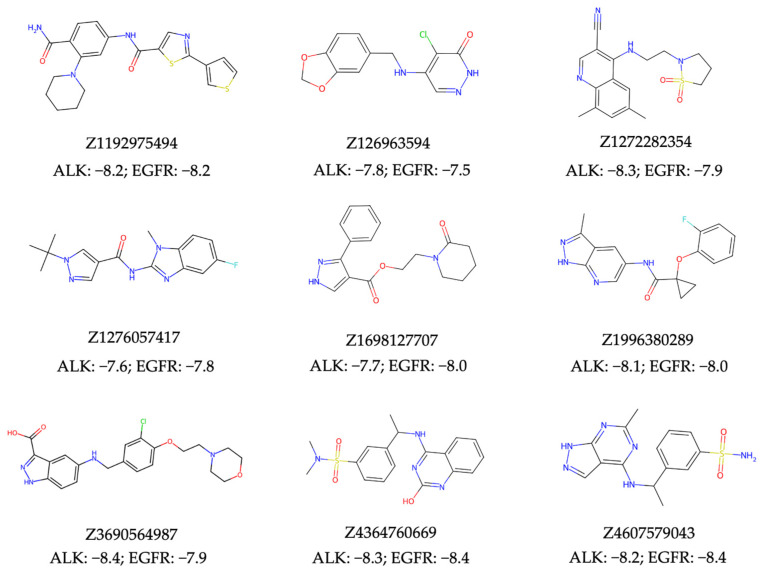
The structures and the top docking scores (kcal/mol) of predicted dual hits with ALK and EGFR.

In evaluating the virtual screening performance of our NAPU-bagging SVM model in discovering new dual ALK-EGFR TKIs from large chemical libraries, we conducted a standard ultra-large virtual ligand screening (VLS) using the 0.46M compounds from the Enamine HLL-460 library. Separate screenings were carried out for both targets, resulting in 650 hits for ALK (Supplementary Table S11), 436 for EGFR (Supplementary Table S12), and 11 intersecting dual hits (Prob. > 0.75). To ensure that the chemical structures of the resulted hits aligned with favorable medicinal chemistry properties, we eliminated all hits containing patterns described by the PAINS [63], Dundee Rule [64], and BMS HTS Deck Filters [65] sets, which denote substructures that could be promiscuously reactive, mutagenic, or pharmacokinetically unfavorable. Additionally, all hits were required to meet Lipinski’s rule of five [66] and the Ghose criteria [67] for “drug-likeness”. This refinement process narrowed down the initial pool of 11 dual hits to 9 of them (Figure 6, Supplementary Table S13).

**Figure 7:**
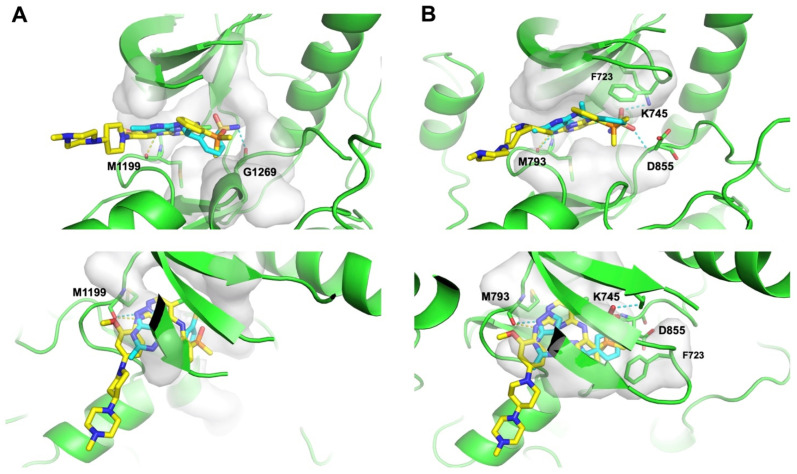
Binding modes of Z4607579043 with (**A**) ALK and (**B**) EGFR in comparison with Brigatinib. Blue: Z4607579043; yellow: Brigatinib; dashed line: hydrogen bond.

Subsequently, we conducted molecular docking to evaluate the binding energetics and modes between the identified dual hits and ALK and EGFR. Specifically, we extracted the kinase domain structures from the complex structures of ALK and EGFR with Brigatinib to perform docking simulations. As a result, all hits exhibited docking scores of less than −7.5 kcal/mol with both targets (Figure 6). Taking Z4607579043 as an example, the docking study revealed its favorable binding pose in both the ALK and EGFR ATP-binding pockets, with respective docking scores of −8.2 kcal/mol and −8.4 kcal/mol, indicating the potential of Z4607579043 as an active dual inhibitor. Comparing its binding mode to that of Brigatinib, Z4607579043 forms hydrogen bonds with the backbone oxygen and amino group of the conserved Met residues of ALK (M1199) and EGFR (M793) in their ATP-binding pockets, a key binding contribution for Brigatinib as a dual inhibitor (Figure 7). Furthermore, Z4607579043 also establishes hydrogen bonds with additional residues in the pockets, specifically G1269 in ALK and K745 and D855 in EGFR (Figure 7), further strengthening the binding affinity. The close proximity of the benzene ring of Z4607579043 to F723 in the EGFR pocket also suggests the possibility of a π−π interaction. For the remaining hits, see Supplementary Figure S1 for their selected docked poses. With the exception of one pair (Z1272282354 binding to EGFR), all exhibited a binding mode analogous to that of Brigatinib. Therefore, we posit that dual hits identified by NAPU-bagging SVM have promising potential as dual ALK-EGFR inhibitors following further optimization.

Worth highlighting is the fact that these dual hits exhibit structural similarities as low as 0.26 with known active TKIs targeting ALK or EGFR (Table 2), suggesting that our approach can detect structurally novel drug hits. These findings collectively demonstrate that our NAPU-bagging SVM method not only effectively identifies potential MTDLs from large compound libraries but also uncovers novel hit molecules in terms of structure.

### 2.6. NAPU-Bagging SVM for Discovering Novel Multitarget Agonists for Dopamine Receptors

Dopamine receptors play a crucial role as drug targets for a variety of central nervous system (CNS) disorders, including Parkinson’s disease [68], schizophrenia [69], and attention deficit hyperactivity disorder (ADHD) [70]. The activation of D1R-D5R dopamine receptors has emerged as a promising therapeutic approach for Parkinson’s disease [71]. However, the development of pan-agonists targeting multiple dopamine receptor family members poses significant challenges. Therefore, we further leveraged our NAPU-bagging SVM models to identify pan-agonists capable of activating D1R-D5R with reduced adverse effects, offering promising therapeutic potential for CNS disorders. This serves as an additional assessment of the broader applicability of this proposed novel strategy.

**Figure 8:**
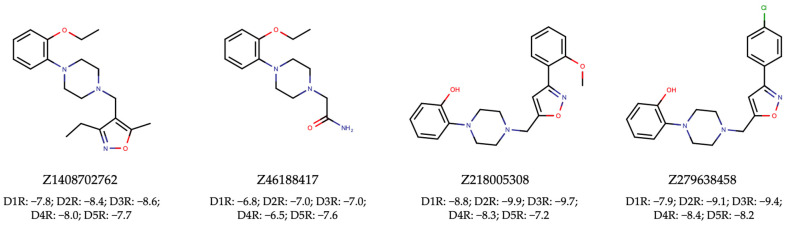
Hit pan-agonists targeting D1R-D5R identified from the Enamine HLL-460 library, along with their respective docking scores (kcal/mol) with D1R-D5R.

To achieve this, we proceeded with VLS using the Enamine HLL-460 library. The resulting hits (Supplementary Tables S14–S18) underwent the same rigorous filtration for unwanted patterns and assessment for drug-likeness as was employed in the discovery of dual ALK-EGFR TKIs (Supplementary Table S19). Ultimately, this meticulous process led to the identification of four pan-hits, as depicted in Figure 8. Subsequent molecular docking analysis revealed that three out of the four pan-hits formed tight interactions with all D1R-D5R targets (Figure 8). Notably, the maximum *Tanimoto* similarity between the active small molecules in the DRD1-DRD5 training set and the four selected active agonists ranged from 0.269 to 0.614 (Table 3). These results once again underscore the effectiveness of our proposed NAPU-bagging SVM approach in the virtual screening and discovery of MTDLs, particularly novel ones.

In a further endeavor to evaluate the predictive efficacy of NAPU-bagging SVM, we examined the activity of rotigotine, the only FDA-approved pan-agonist for dopamine receptors (D1R to D5R) [72]. Remarkably, the NAPU-bagging SVM demonstrated commendable predictive efficacy for four of the five targets (predicted probabilities ranging from 0.754 to 0.996), with the one exception being DRD5 (predicted probability = 0.216). It is important to acknowledge that the limited availability of training data for DRD5 (consisting of only 155 positive samples and 93 negative samples for this specific target) may have impacted the comprehensive understanding of the intricate chemical relationships governing the prediction of drug–target bioactivities. However, in the validation of multitarget drug activity experiments, priority is still given to molecules that exhibit superior activity across most protein subtypes of dopamine receptors. This approach is preferred because such molecules have the potential for subsequent optimization into lead compounds with enhanced efficacy and safety profiles through further molecular modifications.

## 3. Materials and Methods

### 3.1. Datasets for Training, Evaluation, and Virtual Screening

*Training datasets*: Bioactivity data for five tyrosine kinases (ALK, EGFR, FGFR1, MET, and VEGFR1) and one serine/threonine protein kinase (MAPK1) were collected from the ChEMBL database (v29). Their binding affinities were represented using pChEMBL values, which are the negative logarithms of various half-maximal response measurement values (IC50, XC50, EC50, AC50, Ki, Kd, ED50, and potency). Consistent with most compound–protein interaction prediction studies [52], a mixture of these different measurements were used, and a common set of cut-offs were used to partition the data into binary labels. Since there is no unified gold standard for defining actives and inactives, we followed existing studies [73] in defining those with pChEMBL values greater than 7.5(7) as positives and those with values less than 6.5(6) as negatives for binary classifier training of kinase and GPCR, respectively. The compounds with pChEMBL values ranging from 6.5(6) to 7.5(7) were excluded to mitigate the effects of experimental errors [74] and potential mismatches between different measurements.

*Evaluation datasets*: We curated the bioactivity data of the same targets from ChEMBL v32, ExCAPE-DB and DrugBank (version 5.1.10). To prevent the potential biases from data leakage, all training data were excluded to form the external validation datasets. In addition, the Enamine Hit Locator Library (HLL-460), featuring 0.46 million diverse compounds with high MedChem tractability, was utilized for virtual screening of MTDLs. Notably, for virtual screening purposes, the training and validation datasets were merged to retrain the model, ensuring comprehensive coverage of diversity in the screening process.

### 3.2. Data Pools for Augmentation Strategy

To address the class imbalance in the training datasets, two data pools were generated from the PubChem database. Using k-means clustering with PubChem 881 bits fingerprints as input features, a total of 75,745 molecule clusters were formed from the massive pool of 112,428,719 compounds available in PubChem. For each chosen target, a single molecule was randomly from each cluster that did not have a known inhibitor to form the *putative negative* set. Additionally, a single representative molecule was chosen from each cluster to form the *unlabeled* set. To mitigate the parameter tuning complexity of SVM, and to keep the training time within reasonable limits, a random subset of up to 50,000 molecules was sampled from both the *putative negative* and *unlabeled* sets. The *putative negative* set served as negative training examples, while the *unlabeled* set was incorporated into the PU learning framework to enhance the model generalization.

### 3.3. Molecular Representations

We systematically evaluated 14 compound representations in ligand-based virtual screening tasks including 7 sets of topological path-based fingerprints (namely, ECFP4, AtomPairFP, TorsionFP, RDkitFP, AvalonFP, MHFP6, and MAP4), 2 sets of pharmacophore-based fingerprints (PharmacoErGFP and PharmacoPFP), and 3 sets of substructure-key SMARTS-based fingerprints (PubChemFP, MACCSFP, and EstateFP). Thirteen classes of constitutional, physicochemical, and topological descriptors, including Autocorr, InfoContent, Topology, Path, Connectivity, Kappa, Estate, Charge, Matrix, Fragment, Property, Constitution, and MOE (Molecular Operating Environment), were merged as one representation of 1456 descriptors. All of these predefined representations were calculated using Python scripts with the RDKit package (https://www.rdkit.org/, accessed on 1 February 2024).

In addition to these fingerprints and molecular descriptors designed by chemists, we also considered learnable graph representation of compounds, which is widely adopted in deep learning methods, where atoms were converted to nodes and chemical bonds were converted to edges. In this study, one of the state-of-the-art methods, AttentiveFP, was selected for comparison with other representations.

### 3.4. Support Vector Machine (SVM)

The theoretical aspects of SVM have been extensively discussed elsewhere [75]. SVM operates by identifying a hyperplane that maximizes the margin between classes, thereby improving the generalization performance. It is capable of handling both linearly separable and non-linearly separable data by employing diverse kernel functions to map the input data into a higher-dimensional space. In this study, the SVM model was specifically trained to optimize the parameters of the c-Support Vector Classification (c-SVC) with the Radial Basis Function (RBF) kernel. All SVM models were implemented using Python with the scikit-learn package.

### 3.5. DL-Based Sequence-to-Drug Models

To enable a comprehensive comparison between ML and DL methods for drug–target potency prediction, this study incorporated ten prominent DL-based sequence-to-drug models, including DeepDTA [44], DeepConv-DTI [37], TransformerCPI [45], VQA-seq [46], MolTrans [47], GraphDTA [48], MGraphDTA [49], HyperAttentionDTI [50], DrugBAN [51], and TransformerCPI2.0 [52]. These models offer varied protein featurization, compound representation, and network frameworks for potency learning. The suite of models was faithfully re-implemented using the cutting-edge DL frameworks PyTorch 2.2 and PyTorch Lightning. The only modification from the original models was the utilization of up-to-date data to harness the expanding repository of bioassay information.

### 3.6. Molecular Docking

The crystal structures of ALK (PDB id: 6MX8) and EGFR (PDB id: 7ZYM), along with the cryo-EM structures of the D1R-Gs-rotigotine complex (PDB id: 8IRR), D2R-Gi-rotigotine complex (PDB id: 8IRS), D3R-Gi-rotigotine complex (PDB id: 8IRT), D4R-Gi-rotigotine complex (PDB id: 8IRU), and D5R-Gs-rotigotine complex (PDB id: 8IRV), were retrieved from the Protein Data Bank (PDB) and processed using prepare_receptor4 and prepare_ligand4 within AutoDockTools. Prior to molecular docking, the proteins were protonated, and water molecules were removed. During the molecular docking procedure, the receptors were prepared by adding polar hydrogens. We defined the binding sites with grid boxes measuring approximately 20 Å × 20 Å × 20 Å, centered on the crystal ligand. The docking simulations were performed using AutoDock Vina with default settings.

## 4. Conclusions

Drug discovery has traditionally emphasized designing ligands with high selectivity for individual drug targets to mitigate off-target side effects. However, the intricate nature of diseases such as cancer, neurodegenerative disorders, cardiovascular conditions, and infections often involves multiple targets and signaling pathways. In response to this complexity, the development of multitarget agents has gained traction to enhance therapeutic outcomes and safety by simultaneously intervening in multiple receptors. Two primary approaches to multitarget therapeutics are drug combinations and multitarget-directed ligands (MTDLs). While drug combinations offer flexibility in dosing and lower clinical trial costs, they can introduce treatment complexities and adverse effects. In contrast, well-designed MTDLs have shown potential to address these challenges by targeting multiple receptors synergistically to maximize therapeutic benefits while minimizing side effects.

The emergence of machine learning (ML) and deep learning (DL) methods in drug discovery, particularly in MTDL development, has opened new avenues for optimizing virtual screening processes. This study focused on exploring the synergy between ML/DL methodologies and molecular representations, alongside an optimized data augmentation strategy, to enhance the screening of MTDLs. Through meticulous experimentation, we have developed a novel semi-supervised learning framework known as Negative-Augmented PU-bagging (NAPU-bagging) SVM, which serves as a robust tool for managing false positive rates in virtual screening while ensuring high recall rates. This innovative approach involves utilizing bootstrap aggregation to create multiple resampled bags from the training data, where each bag consists of all true positive samples, along with randomly sampled true negative and unlabeled instances at an optimized ratio. An ensemble of SVM classifiers is trained on each bag of samples to distinguish positive samples from true negative and unlabeled ones. The final prediction is aggregated from the collective outputs of the ensemble of classifiers.

The results of this study not only showcase the effectiveness of the NAPU-bagging SVM approach in identifying potential MTDLs from extensive compound libraries but also highlight its capacity to uncover structurally novel hit molecules. This innovative methodology has the potential to transform drug discovery efforts by enhancing the accuracy and efficiency of virtual screening processes. As we navigate the intricate landscape of drug development, the integration of advanced machine learning techniques like NAPU-bagging SVM offers a promising pathway to discover novel therapeutics with enhanced efficacy and safety profiles.

## Figures and Tables

**Table 1:** Performance evaluation of different data augmentation strategies.

Strategy	Metrics	ALK	EGFR	FGFR1	MAPK1	MET	VEGFR1	Average
Baseline SVM	SPE	0.9886	0.9767	0.9961	0.9940	0.9915	0.9743	0.9869
Tao’s strategy		0.9929	0.9881	0.9973	0.9988	0.9931	0.9955	0.9943
PN-weighted		0.9922	0.9881	0.9972	0.9979	0.9923	0.9958	0.9939
PU-Iterative		0.9939	0.9876	0.9976	0.9988	0.9946	0.9952	0.9946
PU-bagging		0.9550	0.9104	0.9354	0.8555	0.9516	0.9275	0.9226
NAPU-bagging		0.9904	0.9695	0.9878	0.988	0.9872	0.9816	0.9841
Baseline SVM	SEN	0.5819	0.6467	0.2048	0.7264	0.7701	0.5806	0.5851
Tao’s strategy		0.4892	0.5923	0.1808	0.4865	0.6848	0.3548	0.4647
PN-weighted		0.4547	0.5923	0.1846	0.5878	0.6588	0.4194	0.4829
PU-Iterative		0.4935	0.6301	0.1858	0.6149	0.7108	0.4516	0.5145
PU-bagging		0.8276	0.9569	0.9456	0.8581	0.9753	0.8387	0.9004
NAPU-bagging		0.6164	0.8243	0.5107	0.7264	0.8640	0.6452	0.6978
Baseline SVM	F1	0.6429	0.694	0.3273	0.2666	0.8235	0.1295	0.4806
Tao’s strategy		0.5997	0.6956	0.2979	0.4318	0.7754	0.2683	0.5114
PN-weighted		0.5642	0.6956	0.3029	0.4065	0.7528	0.3210	0.5072
PU-Iterative		0.6107	0.7217	0.3059	0.5170	0.8008	0.3182	0.5457
PU-bagging		0.6057	0.6856	0.6945	0.0188	0.7653	0.0740	0.4740
NAPU-bagging		0.6810	0.7820	0.6163	0.1587	0.8571	0.1860	0.5469
Baseline SVM	BEDROC	0.8118	0.9185	0.7700	0.7261	0.8984	0.4639	0.7648
Tao’s strategy		0.7480	0.9299	0.8217	0.6107	0.9120	0.5086	0.7552
PN-weighted		0.7180	0.9299	0.8249	0.6607	0.9049	0.5366	0.7625
PU-Iterative		0.8375	0.9400	0.814	0.7518	0.9023	0.5264	0.7953
PU-bagging		0.7900	0.8164	0.8473	0.6013	0.8819	0.5047	0.7403
NAPU-bagging		0.7967	0.9273	0.8134	0.7030	0.9005	0.4943	0.7725

**Table 2:** The maximal *Tanimoto* similarity between the hit dual ALK-EGFR inhibitors and known positive compounds.

ID	ALK	EGFR
Z1192975494	0.304	0.315
Z1272282354	0.287	0.315
Z1276057417	0.360	0.360
Z1698127707	0.268	0.306
Z1996380289	0.373	0.373
Z3690564987	0.344	0.416
Z4364760669	0.260	0.433

Note: *Tanimoto* similarity here means Tanimoto/Jaccard coefficients calculated by RDKit using ECFP fingerprints.

**Table 3:** The maximal *Tanimoto* similarity between the candidate pan-hits and known positive compounds.

ID	DRD1	DRD2	DRD3	DRD4	DRD5
Z1408702762	0.389	0.525	0.580	0.580	0.269
Z218005308	0.308	0.519	0.523	0.554	0.300
Z279638458	0.297	0.511	0.473	0.473	0.282
Z46188417	0.438	0.526	0.614	0.614	0.288

## Data Availability

The data underlying this article are available in the article and in its online Supplementary Materials.

## Supplementary Materials

The following supporting information can be downloaded at: https://www.mdpi.com/article/10.3390/ijms25158239/s1.
