# Supplementary material for: Developing a Semi-Supervised Approach Using a PU-Learning-Based Data Augmentation Strategy for Multitarget Drug Discovery
Source: Int J Mol Sci. 2024 Jul 28;25(15):8239. doi: 10.3390/ijms25158239 (PMC11312053; doi:10.3390/ijms25158239)
Supplement: Supplementary file 1 [file ijms-25-08239-s001.zip › Supplementary File.pdf]

Table S1. Statistics of compound records in collection of ChEMBL, BindingDB and PDBbind.

# of compounds only with positive records (%)	# of compounds with positive and negative records (%)	# of compounds only with negative records (%)	Total
519,157 (62.23%)	9,9192 (11.89%)	215,866 (25.88%)	834,215 (100%)

Table S2. Statistics of target records in collection of ChEMBL, BindingDB and PDBbind

# of targets only with positive records (%)	# of targets with positive and negative records (%)	# of targets only with negative records (%)	Total
1,049 (26.82%)	2,276 (58.19%)	586 (14.98%)	3,911

Table S3. The number of proteins for modeling in each family and the ranges of ratios of positive to negative compounds used for modeling.

Family	# of Proteins	Min Ratio	Max Ratio
**Ion_channel**	19	0.453	73.333
**Kinase**	95	0.005	5.111
**Membrane_receptor**	79	0.037	15.313
**Non_kinase_enzyme**	96	0.000	46.500
**Nuclear_receptor**	13	0.023	2.634
**Others**	46	0.000	99.333

Table S4. The number of experimentally determined and augmented data entries in benchmark datasets.

Training dataset		External evaluation datasets		
Target	ChEMBL v29	ChEMBL v32	ExCAPE	DrugBank
	POS/NEG/PN or UN	POS/NEG	POS/NEG	POS/NEG
ALK	728 / 1,328 / 50,000	275 / 107	182 / 370	7 / 8,854
EGFR	1,561 / 4,709 / 50,000	663 / 590	1,177 / 451	24 / 8,837
FGFR1	597 / 2,020 / 50,000	667 / 239	437 / 111	13 / 8,848
MAPK1	2,287 / 14,454 / 50,000	108 / 91	177 / 174,152	11 / 8,850
MET	1,330 / 2,240 / 50,000	364 / 155	577 / 434	11 / 8,850
VEGFR1	305 / 1,249 / 50,000	14 / 62	-	17 / 8,844

Note: ChEMBL v32 represents data included in ChEMBL version 32 but not in version 29; POS: positive; NEG: negative; PN: putative negative; UN: unlabelled; Compounds with an affinity above a pChEMBL value of 7.5

(equivalent to less than 31.6 nM) were labeled as positive, while those with an affinity below a pChEMBL value of 6.5 (equivalent to more than 316 nM) were labeled as negative. These thresholds were specifically chosen to balance the number of known positives and negatives and were used to develop models capable of identifying highly active compounds.

Table S5. Average rank scores of 14 features across 17 evaluation datasets.

	ACC	BEDROC	FPR	SEN
AtomPairFP	13	12	10	13
AttentiveFP	3	2	1	14
AvalonFP	4	11	9	5
Descriptors	14	8	12	6
ECFP4	12	14	11	12
EstateFP	1	1	2	1
MACCSFP	6	4	4	11
MAP4	5	10	7	3
MHFP6	11	7	14	2
PharmacoErGFP	2	6	3	8
PharmacoPFP	8	3	5	7
PubChemFP	7	5	6	9
RDkitFP	9	13	13	4
TorsionFP	10	9	8	10

Table S6. The number of experimentally determined bioactivity data entries for kinases and their corresponding cluster numbers.

Target	Positive		Negative	
	# of compounds	# of clusters	# of compounds	# of clusters
ALK	1,003	130	1,435	757
EGFR	2,224	311	5,299	1,783
FGFR1	1,264	121	2,131	1,032
MAPK1	2,395	442	14,545	8,687
MET	1,694	261	2,395	1,149
VEGFR1	319	128	1,311	658

Table S7. The number of bioactivity data entries for dopamine receptors and their corresponding cluster numbers.

Target	Positive		Negative		Unlabeled
	# of compounds	# of clusters	# of compounds	# of clusters	
**DRD1**	895	419	775	642	50,000
**DRD2**	3,861	1,253	4,342	3,291	50,000
**DRD3**	3,730	1,126	2,178	1,840	50,000
**DRD4**	1,270	552	488	314	50,000
**DRD5**	155	115	93	77	50,000

Note: The 50,000 unlabeled compounds came from randomly selected 50,000 clusters.

Table S8. The experimental inhibitory activity data for the known ALK-EGFR dual inhibitors.

Compound	Target	IC50 (nM)	Source
**Brigatinib**	ALK	1.7 – 4.9	ChEMBL
	EGFR	1.0 – 203.0	
**9j**	ALK^{WT}	129.81 ± 15	[61]
	EML4-ALK	56.3 ± 0.3	
	$EGFR^{WT}$	236.16 ± 27	
	$EGFR^{L858R/T790M}$	35.7 ± 0.9	
**11**	ALK^{WT}	9.8	[62]
	ALK^{R1275Q}	0.82	
	ALK^{L1196M}	0.59	
	ALK^{F1174L}	0.92	
	ALK^{C1156Y}	1.0	
	$EGFR^{WT}$	108.0	
	$EGFR^{L858R/T790M}$	3.6	
	$EGFR^{T790M}$	3.9	

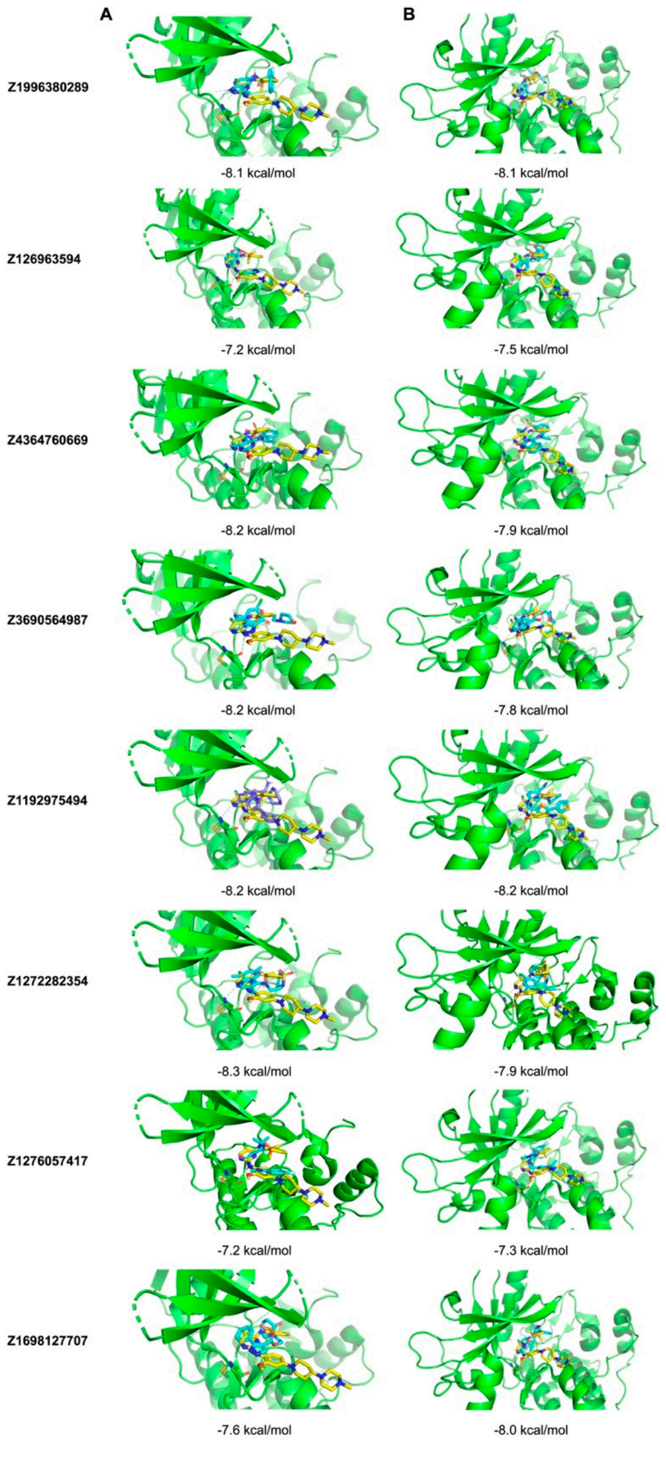

Figure S1. Binding modes of 8 screened compounds with (A) ALK and (B) EGFR in comparison with Brigatinib. Blue: screened compounds; yellow: Brigatinib; dashed line: hydrogen bond. All pairs except Z127228354 – EGFR show similar binding modes to Brigatinib.
